# Efficacy of single dose of an inactivated porcine circovirus type 2 (PCV2) whole-virus vaccine with oil adjuvant in piglets

**DOI:** 10.1186/1751-0147-54-67

**Published:** 2012-11-21

**Authors:** Kun Yang, Wentao Li, Huihui Niu, Weidong Yan, Xiaoli Liu, Yang Wang, Shuang Cheng, Xugang Ku, Qigai He

**Affiliations:** 1 State Key Laboratory of Agricultural Microbiology, Huazhong Agricultural University, Wuhan, 430070, China; 2 Institute of Food Science and Technology, Huazhong Agricultural University, Wuhan, 430070, China

**Keywords:** Porcine circovirus type 2, Post-weaning multisystemic wasting syndrome, Single-dose immunization, Formalin-inactivated vaccine

## Abstract

**Background:**

Post-weaning multisystemic wasting syndrome (PMWS) associated with PCV2 is one of the most costly diseases currently faced by the swine industry. The development of effective vaccines against PCV2 infection has been accepted as an important strategy in the prophylaxis of PMWS.

**Methods:**

In the present study, a PK-15 cell-adapted formalin-inactivated prototype vaccine candidate was prepared using a strain of PCV2 from China. Inactivation of the virus was accomplished using a standard formalin inactivation protocol. The protective properties of the inactivated PCV2 vaccine were evaluated in piglets. Ten 28-day-old pigs were randomly assigned to two groups, each with five. Group 1 was vaccinated intramuscularly with the inactivated virus preparation; Group 2 received sterile PBS as a placebo. By 28 days post-vaccination (DPV), Groups 1 and 2 were challenged intranasally and intramuscularly with 5 × 10^7^ TCID_50_ of a virulent PCV2 isolate.

**Results:**

The vaccinated pigs seroconverted to PCV2 and had high levels of serum antibodies to PCV2 at 28 days after vaccination, whereas the control pigs remained seronegative. No significant signs of clinical disease were recorded following the challenge with PCV2, but moderate amounts of PCV2 antigen were detected in most lymphoid organs of the control pigs. PCV2 was detected in two out of the five vaccinated pigs. Furthermore, pathological lesions and viremia were milder in the vaccinated group.

**Conclusions:**

The obtained results indicate that the inactivated PCV2 virus vaccine with an oil adjuvant induce an immunological response in pigs that appears to provide protection from infection with PCV2. The vaccine, therefore, may have the potential to serve as a vaccine aimed to protect pigs from developing PMWS.

## Background

Post-weaning multisystemic wasting syndrome (PMWS) was first observed in piglets of a high-health herd in western Canada in 1991 [1], and similar disorders were subsequently observed in herds in Europe, the United States and Asia [2-7]. PMWS primarily affects pigs between 5 and 18 weeks of age [8]. The clinical signs of this syndrome include progressive weight loss, unthriftiness, paleness of the skin and dyspnea, and, less frequently, diarrhea and jaundice. Grossly, the main features of PMWS are generalized lymphadenopathy (with the superficial inguinal, submandibular, mesenteric and mediastinal lymph nodes being the most affected) and tan-mottled, non-collapsed lungs [9]. Histopathological findings are characterized by lymphocyte depletion of follicular and interfollicular areas together with macrophage infiltration of lymphoid tissues, interstitial pneumonia, hepatitis and nephritis [9]. The mortality rate may vary from 1 to 2% up to 30% in complicated cases [5]. The accumulated evidence indicates that PCV2 is the major pathogen in the etiology of PMWS [10-13]. Moreover, PCV2 has also been associated with other swine diseases, such as porcine dermatitis and nephropathy syndrome (PDNS), porcine respiratory disease complex (PRDC) and reproductive failure, which are now collectively referred to as PCVAD or PCV2-associated diseases, a name that the American Association of Swine Veterinarians (AASV) uses to group together all the diseases attributed to PCV2, including PMWS [14]. PCV2 is a non-enveloped, single-stranded, circular DNA virus with a 1.76-kb ambisense genome [15]. The PCV2 genome is assumed to have 11 potential open reading frames (ORF1-11) [16]. Previous studies have demonstrated that ORF1, -2 and −3 encode a 35.7 kDa replication (Rep) protein involved in virus replication [17], a 27.8 kDa capsid (Cap) protein involved in PCV2 immunogenicity [18-20] and a protein involved in PCV2-induced apoptosis [21], respectively. Immunization against PCV2 has been studied intensely and found to be the most effective strategy for protecting pigs against PCV2 infection. Over the years, various PCV2 vaccines, including DNA vaccines [22,23], chimeric PCV1-2 vaccines [24] and subunit vaccines [25], have been described for the control of PCV2 infections. Although both DNA vaccination and subunit vaccination based on the ORF2 gene have shown relatively satisfactory immunization against PCV2, DNA vaccination is not suitable for clinical application because of the possibility of carcinogenesis [26]. Subunit vaccination is safer but not suitable for farmers in developing countries due to its high cost. Inactivated vaccines are theoretically advantageous because they represent a complex mixture of viral antigens closely resembling the native virion. Formalin is known to interact and induce the cross-linking of viral proteins, leading to the loss of virus infectivity. The formalin inactivation of a virus has been successfully used to develop safe and efficacious human and veterinary vaccines since 1955 [27]. The use of formalin inactivation for virus vaccine development is attractive from a safety perspective as the virus cannot revert to a virulent form, because there is no virus replication during immunization. The use of formalin to inactivate viruses is also attractive from a manufacturing perspective as the inactivation process is relatively simple to develop.

Therefore, we prepared a PK-15 cell culture-derived, formalin-inactivated prototype vaccine candidate for PCV2. Animal experiments were designed to evaluate the immunogenicity and efficacy of the inactivated PCV2 vaccine in combination with an oil adjuvant in conventional pigs following intramuscular (IM) administration. The protective efficacy of the immunological response was evaluated by challenge with PCV2 via the intranasal (IN) and intramuscular (IM) routes.

## Methods

### Virus

The WuHan strain of PCV2 (GenBank: FJ 598044) was used to prepare both the vaccine and the challenge inoculum for the animals. The virus was propagated in porcine kidney cells (PK-15) free of PCV1 contamination and grown in Minimal Essential Medium (MEM) containing 10% fetal bovine serum. The titer of the virus stock was 10^7^ TCID_50_/ml.

### Vaccine preparation

The PCV2 was produced according to the methods described by Tischer et al. [28], with minor modifications. Briefly, the PCV2 WuHan strain was inoculated into PK-15 cells by one-step inoculation and incubated at 37 °C with 5% CO_2_ in MEM containing 10% fetal bovine serum. One day after seeding, the medium was removed, and the cultures were incubated with 300 mM D-glucosamine in Hank^’^s salt solution (HSS) for 30 min at 37°C in a volume sufficient to cover the monolayer. Thereafter, the treated PK-15 cell cultures were incubated in growth medium for two additional days in MEM containing 2% fetal bovine serum at 37°C with 5% CO_2_. The inoculated cells were frozen and thawed three times. After centrifugation at 5000 × *g* for 20 min, the supernatant was titered and then inactivated by incubation with formalin at a 0.3% final concentration for 48 hours at 37°C. Virus inactivation was confirmed by the inoculation of the formalin-treated samples into PK-15 cells. The formalin in the samples was then neutralized by the addition of one part 0.2% (w/v) sodium metabisulfite to 100 parts of vaccine. The titer of the virus suspension prior to inactivation was 10^7^ TCID_50_/ml. The inactivated viral suspension was then mixed with an oil adjuvant (MARCOL 52, EXXonMobile, USA) at an appropriate ratio.

### Animals and housing

Ten healthy, weaned, three-week-old crossbred piglets were obtained from a pig farm that was negative for PCV2 and PRRSV infections. The selected animals were transported to the animal facilities at the Huazhong Agricultural University and allowed to acclimatize for seven days before the PCV2 vaccination. All experimental protocols were approved by the Research Ethics Committee of College of Veterinary Medicine, Huazhong Agricultural University, Hubei, China (No. 2009–0012). The animals were randomized into two groups with five pigs each and raised separately in two isolation rooms with individual ventilation. The pigs were provided commercial diets and water ad libitum. During that period, the animals were weighed and bled to assess their serological and virological status to PCV2, and all piglets were confirmed to have neither serological nor virological evidence of previous exposure to PCV2 prior to the vaccination (data not shown).

### Experimental design and sample collection

After arriving at the animal facilities, the piglets were given an identity and distributed into two groups with stratification by their weight, namely Group 1 and Group 2. The animal care was provided under an Institutional Animal Care and Use Committee approved protocol. At 4 weeks of age, each piglet in Group 1 was intramuscularly injected with 2.0 ml of prepared inactivated PCV2 vaccine on the right side of the neck, whereas each piglet in Group 2 received 2.0 ml of sterile PBS as a placebo. Next, four weeks after the immunization (8 weeks of age), all pigs were challenged with 5 ml (2.5 ml intranasally and 2.5 ml intramuscularly) of the WuHan strain of PCV2 at a dose of 10^7^ TCID_50_/ml. After the inoculation, the pigs were monitored for 28 days. During this period, the pigs were clinically examined, and rectal temperatures were recorded on a daily basis. Body weight was measured before the immunization, at challenge and at the time of necropsy. The relative daily weight gains (expressed as daily weight gains/primary body weight, RDWG) were determined. Blood samples were collected from the vena cava at the time of the vaccination and at weeks 1, 2 and 3 post-vaccination (PV), at the time of challenge and on a weekly basis thereafter. Sera were obtained and stored at −80°C until the serological and virological test were performed. Twenty-eight days after the challenge, the animals were euthanized with an intravenous overdose of sodium pentobarbital, and a complete necropsy was performed. All tissue collection procedures were performed according to the protocols approved by the Hubei Province PR China for Biological Studies Animal Care and Use Committee. Macroscopic and microscopic lesions were compared between the groups. The amount of PCV2 antigen in the lymphoid tissues was determined by immunohistochemistry (IHC).

### Quantitative real-time PCR for evaluation of viremia

DNA extraction from the serum samples collected on the day of challenge and on DPC 7, 14, 21 and 28 was performed using the E.Z.N.A.^TM^ Viral DNA Kit (OMEGA, USA) according to the manufacturer’s instructions. The DNA was used to quantify the PCV2 genomic DNA copy numbers by real-time PCR. GenBank entry FJ598044 was used for the primer and probe design. The Cap gene region (corresponding to nucleotides 1033–1734 bp of the whole PCV2 genome) [19,29,30] was chosen for the primer and probe design because it has a lower nucleotide homology with porcine circovirus type 1 (PCV1) than ORF1 (~65%) [31]. The forward (5'-CCAGGAGGGCGTTCTGACT-3') and reverse (5'-CGTTACCGCTGGAGAAGGAA-3') primers and probe (5'-AATGGCATCTTCAACACCCGCCTCT-3') were designed using the Primer Express v.1.5 software (ABI Prism 7500 User’s Guide, Applied Biosystems, Foster City, CA, USA). The primers and probe were selected to work under universal conditions (ABI Prism 7500 User’s Guide, Applied Biosystems, USA). The probe was labeled on the 5' end with FAM^TM^ (6-carboxyfluorescein) and on the 3' end with TAMRA^TM^ (6-carboxytetramethylrhodamine). The PCR reaction contained a final concentration of 1× THUNDERBIRD Probe qPCR Mix (TOYOBO, Japan), 0.3 μM each primer, 0.2 μM Taqman Probe, 0.04 μl of 50× ROX reference dye and DNA equivalent to that of 1 ml serum as the template. Autoclaved nanopure water was added to bring the final volume to 25 μl. All reactions were conducted in triplicate on an ABI7500 (Applied Biosystems). The PCR program consisted of one cycle of 95°C for 1 min and 40 cycles of 95°C for 15 s and 60°C for 1 min. For a standard curve, serial dilutions of plasmid pORF2 (the ORF2 gene cloned into the pMD^TM^ 18-T Vector) were used to quantify the virus genomic copy number. The numbers of virus copies for each sample were presented as the mean value of triplicate reactions.

### Serological tests

Cap protein-specific antibodies were determined with an endpoint ELISA using the recombinant Cap protein antigen as described previously [32]. The titers were expressed as the reciprocal of the highest dilution of sera producing ratio values of 2:1.

### Histopathology

Formalin-fixed, paraffin-embedded tissue samples were cut into 4 μm thick slices, stained with hematoxylin-eosin and examined for lesions compatible with PMWS. The sections for histopathological examination were taken from lung and lymphoid tissues, including the lymph nodes (mesenteric and superficial inguinal), tonsil, and spleen. The tissues were examined in a blinded fashion and given a subjective score for the severity of the lesions. The lung scores ranged from 0 (normal) to 3 (severe lymphohistiocytic interstitial pneumonia). Lymphoid tissues were evaluated for the presence of lymphoid depletion, ranging from 0 (normal) to 3 (severe), and for histiocytic inflammation and the replacement of follicles, ranging from 0 (normal) to 3 (severe) [32]. The overall microscopic lymphoid lesion score was the average of the five studied tissues (lung, tonsil, spleen, and the mesenteric and superficial inguinal lymph nodes).

### Immunohistochemistry

IHC for the detection of PCV2-specific antigens was performed on lymphoid tissues, including lymph nodes (mesenteric and superficial inguinal), tonsil, and spleen collected during the necropsy at 28 DPC. A rabbit polyclonal antiserum against PCV2 was used for IHC following procedures described previously [33]. The amount of PCV2 antigen distributed in the tissues was scored in a blinded fashion by assigning a score ranging from 0 for no signal to 3 for a strong positive signal. The mean group score was determined for each tissue and compared between groups.

### Statistical analysis

The statistical analysis was performed by one-way analysis of variance using SPSS version 17.0 (SPSS). The results were considered to be statistically significant for *P* < 0.05.

## Results

### Clinical presentation

None of the animals developed clinical signs compatible with PMWS during the entire experiment. The number of days in which the rectal temperature exceeded 40.0°C and the relative daily weight gain (expressed as the daily weight gain/primary body weight, RDWG) were recorded. As shown in Table 1, no significant differences in the body weight or rectal temperature were observed between the groups at any of the sampling times.

**Table 1:** Clinical signs and weight gain of pigs following intranasal and intramuscular inoculations with PCV2

**Groups**	**Days with fever ( ≥40°C)**	**Body weight**		**Relative daily weight gain**
		**At challenge**	**28 days pc**	
Vaccinated group	0	14.98 ± 1.09	27.96 ± 0.84	0.0311 ± 0.0035
Control group	0.40 ± 0.55	15.16 ± 1.32	27.12 ± 0.90	0.0284 ± 0.0036

### Incidence and amount of PCV2 DNA in serum

PCV2 DNA was not detected in any of the serum samples on the day of the challenge. The log transformed group mean amounts of PCV2 DNA for both groups are summarized in Table 2. In the challenge-control group, all pigs had PCV2 viremia, which persisted for at least 28 days. However, in the vaccination-challenge group three out of five pigs had PCV2 viremia that persisted for 21 days after the PCV2 challenge. At 28 days after the PCV2 challenge, no viremia was observed. In addition, compared with the challenge-control pigs, those in the vaccination-challenge group exhibited a reduction in the level of viremia, although this difference was not significant. This result indicated that the viremia presented in the vaccination-challenge group was lighter than that in the challenge-control group.

**Table 2:** **PCV2 prevalence in the serum of pigs following intranasal and intramuscular inoculations with PCV2, and the mean viral load of the PCV2-positive pigs (log**_**10**_**)**

**Groups**	**Day post-challenge**			
	**7**	**17**	**21**	**28**
**PCV2 in serum**				
Vaccinated group	3 of 5	3 of 5	2 of 5	0 of 5
Control group	5 of 5	5 of 5	5 of 5	5 of 5
**Viral load in positive pigs**				
Vaccinated group	7.69 ± 0.11	7.67 ± 0.22	7.76 ± 0.26	0.00 ± 0.00
Control group	8.31 ± 0.40	8.41 ± 0.91	8.77 ± 0.55	7.72 ± 0.55

### Humoral response

**Figure 1:**
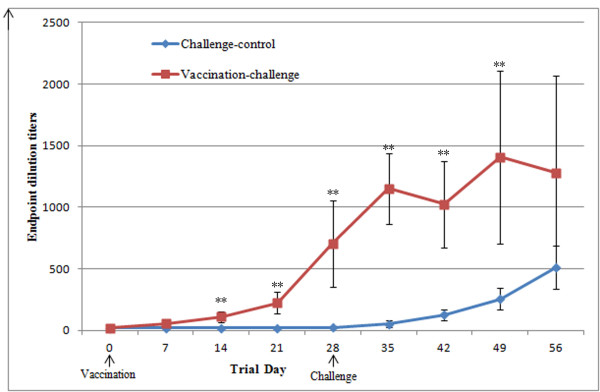
**PCV2-specific antibody detection by ELISA.** ELISA assay for PCV2-specific antibodies in the serum of vaccinated and non-vaccinated pigs from day 28 pre-challenge to day 28 post-challenge. (**) Indicates significantly (P < 0.01) higher PCV2-specific antibody titers in the vaccinated pigs than in the challenge control pigs.

The evolution of the ELISA titers to PCV2 in the vaccinated and non-vaccinated pigs is displayed in Figure 1. Both groups were seronegative for PCV2 when vaccinated on day 0. At the time of the challenge, which was 28 days later, the vaccinated pigs had seroconverted to PCV2 and had significantly (p < 0.05) higher titers of antibodies against PCV2 than the control pigs. Both groups had increased levels of serum antibodies against PCV2 following the challenge, and the non-vaccinated control pigs seroconverted to PCV2 between 14 and 28 days post-challenge (days 42 to 56).

### Histopathological lesions

**Figure 2:**
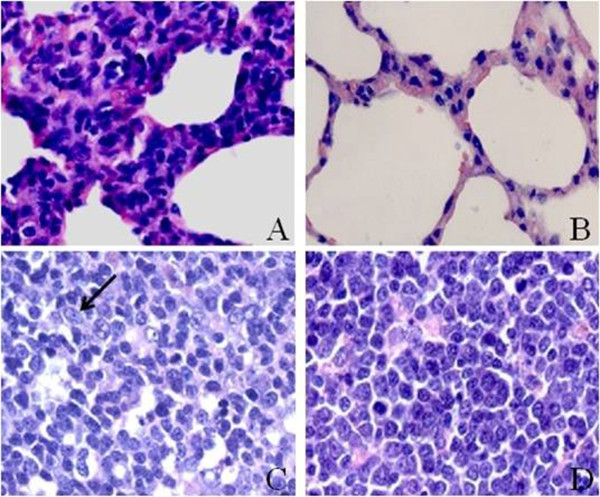
**Histological lesions found in the PCV2-challenged pigs.** Severely thicker alveolus walls were examined in the lung of pigs in the challenge-control group (**A**). Severe infiltration with epithelium-like macrophages (↑) was examined in the lymph nodes of pigs in the challenge-control group (**C**). There were no clear histological lesions in the lungs (**B**) or lymph nodes (**D**) in four out of the five vaccinated pigs.

To investigate the protective efficacy of the inactivated PCV2 vaccine, we compared the pathological changes of the lung and lymphoid tissues between the groups. All pigs in the control group had mildly to moderately thicker alveolus walls (Figure 2A) in their lungs on day 28 post-challenge (day 56), with a total score of 9. These pigs also had mild to moderate histiocytic replacement in the lymphoid tissues (Figure 2C), with a total score of 8. In contrast, no signs of disease were recorded in four of the five vaccinated pigs (Figure 2B and 2D). The fifth vaccinated pig had a score of 1 in the lungs and 1 in the lymphoid tissues.

### Detection of the PCV2 antigen in tissues

At necropsy (DPC 28), the incidences of the PCV2 antigen as determined by IHC for the immunized and non-vaccinated pigs, respectively, were as follows: 2/5 and 5/5 in the superficial inguinal lymph node sections, 2/5 and 5/5 in the mesenteric lymph node sections, 1/5 and 4/5 in the tonsil sections, and 1/5 and 3/5 in the spleen sections (Table 3).The amounts of PCV2 antigen in the immunized pigs were significantly lower than in the non-vaccinated pigs in the superficial inguinal lymph nodes and mesenteric lymph nodes (*P* < 0.01). In addition, compared with the challenge-control group pigs, those in the vaccination-challenge group exhibited a reduction in the amounts of PCV2 antigen in the tonsils and spleen, although these differences were not significant.

**Table 3:** Demonstration of PCV2 by immunohistochemistry at the necropsy performed 28 days after the intranasal and intramuscular inoculations of the pigs with PCV2, shown as the number of PCV2-positive pigs and mean scores

**Groups**		**Body organ**		
	**Superficial inguinal lymph nodes**	**Mesenteric lymph nodes**	**Tonsils**	**Spleen**
**Presence of PCV2**				
Vaccinated group	2 of 5	2 of 5	1 of 5	1 of 5
Control group	5 of 5	5 of 5	4 of 5	3 of 5
**Mean score**				
Vaccinated group	0.40 ± 0.55	0.40 ± 0.55	0.20 ± 0.45	0.20 ± 0.45
Control group	2.40 ± 0.55*	2.00 ± 0.71*	1.00 ± 0.71	0.80 ± 0.84

Values in parentheses are the mean estimated amounts of the PCV2 antigen in lymphoid tissues (range: 0, no antigen detected; 3, high amounts of antigen). * indicates a difference in the mean value scores between the groups (*P* < 0.05).

## Discussion

Post-weaning multisystemic wasting syndrome (PMWS) is recognized as a major disease problem of economic importance in many pig-producing areas of the world [5,8]. It can cause significant levels of mortality in many herds [5]. Although other co-factors have been reported to contribute to this disease, there is no doubt that the expression of the clinical disease is dependent on the presence of PCV2 [34,35]. The development of effective vaccines against PCV2 infection has been accepted as a strategy for the prophylaxis of PMWS. Maternal antibodies against PCV2 have been shown to be present in 3-week-old pigs, which gradually decrease to a very low level in 11-week-old pigs [36]. The low levels of antibodies against PCV2 ensure that the pigs are unable to handle a PCV2 infection, leading them to develop PMWS [37,38]. To create a viable PCV2 vaccine approach, a vaccination method should be designed to induce immunity in piglets prior to the time-point when the weaning maternal immunity makes the piglets susceptible to PCV2 infection. The lack of a consistent, precise and reproducible model of PMWS [14] is one of the main drawbacks for the experimental evaluation of PCV2 vaccines. Therefore, the assessment of vaccine efficacy in terms of protection against clinical disease was not feasible. This situation applies not only to this study but also to earlier ones in which only subclinical infections were developed [24,39-41]. As performed in those studies, the vaccine efficacy may be validated by evaluating some parameters related to PCV2 infection, such as viremia, the presence of microscopic PMWS-like lesions and the viral load in tissues, or by determining the ability of the vaccine to induce an immune response.

Reduced PCV2 viremia was observed in PCV2 vaccine studies in PCV2-negative pigs under experimental conditions [39,41,42]. In the present study, the experimental model using PCV2 alone as an inoculum did not succeed in causing PMWS; however, we examined the PCV2 levels in the sera of pigs by real-time PCR at different times after the challenge, and the results indicated that the proportion of viremic pigs, the days with viremia and the viremia levels in the serum were significantly reduced in the vaccination-challenge pigs compared with their challenge-control counterparts. Additionally, the PCV2 antigen was detected in low-to-high amounts in lymph node, tonsil, and spleen tissues of the non-vaccinated pigs but not those of the vaccinated pigs, with the exception of one pig. This observation suggests that the inactivated PCV2 vaccine can partially prevent PCV2 viremia and significantly reduce the amount of PCV2 virus in the lymphoid tissues, which are important factors in the pathogenesis of PCV2-associated diseases [36,43]. PCV2-specific antibodies have been generally accepted to be associated with protection because field evidence has suggested that the decrease in antibodies contributes to the development of PMWS [2,8,44]. In this study, the ELISA results showed that the level of antibodies against PCV2 in the serum of vaccinated pigs gradually increased and that the seroconversion to PCV2-specific antibodies was detected in two pigs at 14 DPV and all five pigs by 21 DPV. These results confirmed that the inactivated PCV2 whole-virus vaccine with an oil adjuvant could elicit a high level of humoral immune response in swine. The PCV2-challenge in this study was performed using both intramuscular and intranasal routes, which is in contrast to other groups that used the intranasal route only [45,46]. The combination of the intranasal and intramuscular routes of inoculation was found to be successful in obtaining a uniform PCV2 infection level in the pigs [47-49]. We chose to perform both because of the increased likelihood that not all the pigs inhale an equal amount of inoculum due to sneezing and/or labored breathing at the time of inoculation when using only the intranasal route. This approach was performed to ensure that all pigs received a certain amount of the virus and to ensure that the infection was successful. The mean scores of the microscopic lesions in the lung, lymph node, spleen, and tonsil tissues of the vaccinated groups showed that the lesions were less severe (*P* < 0.05) than those of the non-vaccinated group. These results indicate that the inactivated PCV2 vaccine may be effective in protecting pigs from PCV2-associated diseases, but further studies are required to ensure the efficacy of the vaccine.

## Conclusions

In conclusion, the results obtained indicate that an inactivated PCV2-virus vaccine with an oil adjuvant induces an immunological response in pigs that appears to protect the pigs from PCV2 infection. The vaccine may thus have the potential to serve as a vaccine to protect pigs from the development of PMWS.

## Competing interests

None of the authors of this paper have a financial or personal relationship with other people or organizations that could inappropriately influence or bias the content of this paper.

## Authors’ contributions

HQG, YK, and LWT initiated and designed the study; YWD prepared the virus and vaccine; and YK, LWT, NHH, LXL, WY, CS, and KXG performed the experimental infection. All authors were involved in the interpretation of the results and drawing of the conclusions and provided helpful advice in writing the paper. All authors read and approved the final manuscript.
